# Harnessing RNAi nanomedicine for precision therapy

**DOI:** 10.1186/2052-8426-2-5

**Published:** 2014-02-05

**Authors:** Dan Peer

**Affiliations:** Laboratory of NanoMedicine, Department of Cell Research and Immunology, George S. Wise Faculty of Life Science, Tel Aviv, 69978 Israel; Department of Materials Science and Engineering, Faculty of Engineering, Tel Aviv, 69978 Israel; Center for Nanoscience and Nanotechnology, Tel Aviv University, Tel Aviv, 69978 Israel

**Keywords:** RNAi, siRNAs, Precision medicine, Nanomedicine, Targeted Nanoparticles

## Abstract

Utilizing RNA interference as an innovative therapeutic strategy has an immense likelihood to generate novel concepts in precision medicine. Several clinical trials are on the way with some positive initial results. Yet, targeting of RNAi payloads such as small interfering RNAs (siRNAs), microRNA (miR) mimetic or anti-miR (antagomirs) into specific cell types remains a challenge. Major attempts are done for developing nano-sized carriers that could overcome systemic, local and cellular barriers. This progress report will focus on the recent advances in the RNAi world, detailing strategies of systemic passive tissue targeting and active cellular targeting, which is often considered as the holy grail of drug delivery.

## Review

### Introduction

RNA interference (RNAi) is an innate cell machinery for regulation of gene expression. This process is preformed by double-stranded ribonucleic acid (dsRNA) that down-regulates the expression of precise genes with matching nucleotide sequences by decaying specific messenger RNA (mRNA) or by physically obstructing mRNA translation. RNAi can be triggered exogenously by uttering short hairpin RNA (shRNA) with virus-like vectors, or by integrating artificial small interfering RNAs (siRNAs) sequences straight into the cell cytoplasm [1–3].

siRNA is a chemically synthesized double stranded RNA (dsRNA) of 19–21 base pairs with 2-nucleotides unpaired in the 5′-phosphorylated ends and unphosphorylated 3′-ends [3–5]. Inside the cell, siRNAs are included into a protein-RNA complex termed rna induced silencing complex (RISC) that segregate the strands of the RNA duplex and junks the passenger strand also known as the sense strand. The antisense strand then guides RISC to anneal and cut the specific mRNA or inhibits its translation [2, 3]. Through reprocessing the target mRNA, the RISC complex includes the anti-sense strand. This process may have a gene knockdown affect that last up to seven days in dividing cells and numerous weeks in non-dividing cells [3, 6]. Moreover, multiple injections of siRNAs can result in steady therapeutic gene silencing [7, 8].

The combination of silencing any preferred gene and the ability to treat various diseases by addressing potential non-druggable targets coupled with an improved safety and the reduced likelihood for meddling to the endogenous microRNA machinery, emphasizes the potential of RNAi to become a new potential modality for precision medicine.

Aside from this potential, employing RNAi molecules as therapeutics is not a marginal assignment. For instance, for siRNAs, the ~ 13.5 kDa and the net negative charge with more than 40 phosphate groups, makes these molecules an obstacle for crossing [9] the plasma membrane and efficiently entering the cell cytoplasm [2, 3, 10]. Intravenous administration of these molecules causes rapid renal clearance and these molecules are susceptible to RNA degradation by RNAses. In addition, naked siRNAs, which are not chemically modified, could be recognized by special receptors of the immune systems that sense nucleic acids termed Toll-Like Receptors (TLRs) [3, 9]. The binding of siRNAs to these receptors may activate the immune system and provoke different responses such as trigger an interferon response, activate the complement system, and induce cytokines. In addition to immune stimulation, these effects can globally inhibit gene expression and generate off-target properties [9–11]. Hence, the need for selective delivery strategies carrying RNAi payloads is essential. This report will focus on the progress of this field, concentrating mainly on systemic applications of siRNA therapeutics. Special highlight will be made on strategies for therapeutic gene silencing in leukocytes in an era of personalized medicine. In precision and personalized medicine, sequencing of the transcriptome of a diseased individual becomes a reality. Thus, the option to design sequence-specific molecules that can interfere with translation of any given protein can be used to manipulate cellular functions and ultimately, might soon become a new therapeutic modality [12, 13].

### Cellular siRNA delivery strategies

Common strategies used for delivery of RNAi molecules (siRNA, miRNA mimic or anti-miRNAs) for cellular applications are utilizing conventional transfection techniques. Studies with purely physical methods for example electroporation, gene gun or microinjections [14–17], in addition to studies used calcium phosphate [18], commercial cationic reagents both synthetic and natural [4, 19–24] and cell penetrating peptides (CPPs) [25–29], have been efficiently employed to silence specific genes of interest. Beside for the physical methods, namely electroporation and microinjection, all the other approaches share a common denominator – a cationic charge that enables complexation of the negatively charge RNAi payloads and interact with the negatively charged plasma membrane [30, 31]. In this manner, it is important to note that there are evidences for toxicities of cationic lipids and polymers ([32] and reviewed in [33–35]).

The use of RNAi strategies to silence genes of interest *in vitro* is an essential tool for studying gene expression in normal and diseased validating new therapeutic targets [36, 37]. Yet, the ability to manipulate gene function in vivo in a disease model combined with the potential to induce therapeutic gene silencing, may open new avenues for exploiting RNAi as a novel therapeutic modality and ultimately may brings closer the era of personalized medicine when transcriptom of patients will be used in order to tailor a specific RNAi drug to their current disease [13, 30].

Although there are large numbers of technologies available for *in vitro* RNAi delivery shortly represented above, there are still numerous challenges for translating these strategies into clinical practice. The biggest hurdle confronting the translation of siRNAs therapeutic potential into the clinic is the ability to deliver these payloads in a safe- and cell-specific manner [13, 30].

### Local and systemic siRNA delivery strategies

Native delivery of siRNAs has been shown in different disease models [6, 28, 30, 38, 39] and a few current clinical trials are utilizing local and topical delivery strategies [2, 9]. Topical and local administration of polymer-free siRNAs or siRNAs that are complexed with lipids or polymers are utilized for local administration. However, this strategy is mainly applicable for subcutaneous tissues, ocular applications and mucosal surfaces.

Systemic delivery of RNAi molecules is currently considered as the ‘Holy Grail’ of the field. Whereas topical and local RNAi delivery strategies have to employ internalization strategies and endosomal release approaches, systemic RNAi delivery strategies need to really on methods to evade the immune system, frame-up within small blood capillaries, uptake by cells of the mononuclear phagocytic system (MPS), degradation by RNAses, renal clearance, anatomical barriers (such as the liver), immune activation, extravasation from blood vessels to target tissues, and permeation within the tissue to name a few [30].

Naked siRNAs can be delivered systemically via hydrodynamic injection. This strategy, with an unknown mechanism, involves rapid intravenous administration of a large volume of siRNAs in physiologic solutions (about 10% of the body weight administered within 5–10 seconds) [30, 40, 41]. Hepatocytes are taken up these siRNAs molecules using this strategy. Several studies were reported to use successfully this strategy, showing efficient gene silencing in the liver [30, 40–43]. Nevertheless, due to volume overload side effects, the hydrodynamic injection approach is not appropriate for therapeutic applications and was only utilized as a proof-of-concept approach [3].

Naked siRNAs can be employed for kidney targeting. Naked siRNAs are known to be eliminated by the glomerulus, which secrete molecules with weights that are below 40 kDa. Free siRNAs are accumulated in the kidney around 30–40 times more than in any other organ [2, 3]. This feature could be utilized for RNAi therapy, but could also introduce novel hurdles in the form of kidney toxicity. Studies performed in rat models of renal injury showed a therapeutic benefit of silencing the proapoptotic gene, p53 that resulted in renal protection, upon single and multiple administrations [3, 44, 45]. QPI-1002 developed by Quark Pharmaceuticals for acute renal injury and delayed graft function is currently under clinical evaluation [3, 45].

Utilizing naked siRNAs for systemic application is appropriate only when the target organ is the kidney. Else, delivery of siRNA payloads for systemic applications must relay on nano-scale delivery platforms. These nano-scale delivery platforms have to be made from materials that are degradable, non-immunogenic and compatible to the biological environment. In addition, the ability to direct these delivery platforms to specific organs and cells will provide them with additional advantage since only diseased cells will be targeted.

The parental siRNA delivery strategies can be generally divided into two categories: passive tissue and active cellular-based deliveries. While, passive tissue delivery strategies use the propensity of macromolecules and nanoparticles to accumulate in organs of the MPS. The MPS, an integral part of the immune system, consists of phagocytic cells located in reticular connective tissue, primarily monocytes and macrophages [30, 34]. Cells of the MPS reside in the spleen, liver (kupffer cells) and different lymph nodes and take up materials and particles supposed to viruses, parasites and bacteria of different types, sizes, geometry, surface curvature and charge [30, 46]. Thus, many efforts are still focusing on generating safe delivery strategies targeting hepatocytes. Active, cellular-based RNAi delivery strategies are based on specific natural ligands or ligand mimic that guide the nano-carriers to organs, tissues and subsets of cells [30, 47].

#### *Systemic passive delivery strategies*

**Figure 1:**
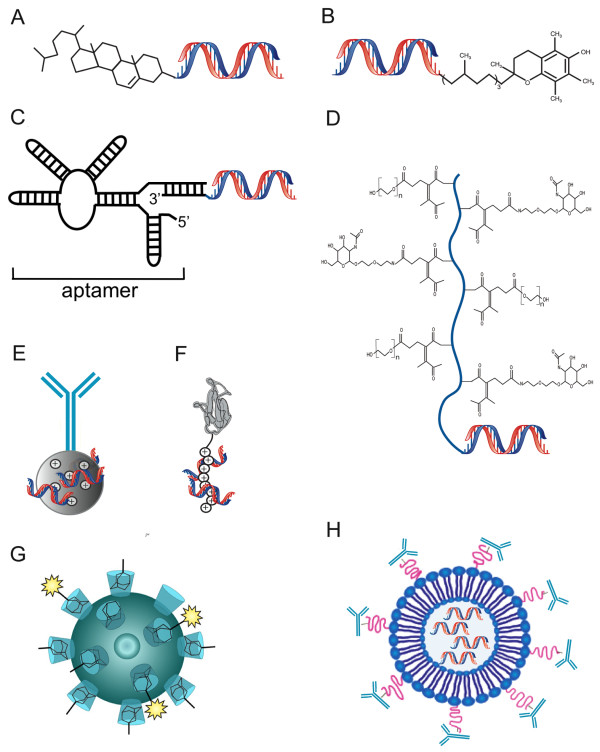
**RNAi delivery systems in development stages include RNAi coupled to cell penetrating molecules such as cholesterol (A); or other low molecular weight molecules (B); conjugated to an aptamer that target a specific cell surface receptor (C); conjugated to cell membrane-penetrating polymer that is linked to targeting small molecules (D), complexed with fusion proteins composed of targeting peptide or a fragment of an antibody coupled to an RNA binding domain that is either protamine (E); or poly-arginine (F); or entrapped within nanoparticles (G); or LNPs (H); bearing targeting moieties.** Reprinted with permission from ref. 31 Copyright 2011 Gene Therapy.

Stable nucleic acid-lipid particles (SNALP) (Figure 1) are ~ 90 nm in diameter lipid-based particles forming liposomes with amino lipid content that encapsulates RNAi payloads. These amino lipids have lipid pKa of 6.4-6.8 and thus are positively charged in low pH. RNAi payloads such as siRNAs are suspended in acetate buffer pH 4.0 and mixed with the lipids that are dissolved in ethanol to form stable nucleic acid-lipid particles [48]. In addition, these particles compose of a diffusible lipid that is conjugated to polyethylene glycol (lipid-PEG) [49, 50]. This unique lipid coating stabilizes the particle in the process of particle creation and provides a neutral surface charge and hydrophilic coating that endow these particles with long circulation properties and reduction in the clearance from the bloodstream. This special liposomal features that combining fusogenic and amino lipids allow for internalization of the particles and efficient release of the RNAi payloads that induce gene silencing of the desired gene. A distribution study showed that around 30% of the siRNAs carried by these particles were accumulated in the liver (with minimal accumulation (0.3%) in the lungs. SNALPs encapsulated ApoB -siRNA has shown significant reduction in the mRNA levels of this gene. In spite of the use of amino lipids in the SNALP formulations that are known to trigger different types of toxicities [33], in a non-human primates toxicity study no adverse effects except slight elevation of liver enzyme release was observed. Based on these studies, several human trials are conducted these days to examine the potency of these particles to deliver specific RNAi payloads for reducing cholesterol levels and as novel therapeutics in liver cancer [2, 45]. This strategy was also used to deliver siRNAs against the polymerase gene of the Zaire strain of Ebola virus and shown to protect guinea pigs from lethal challenge this virus [51]. Different formulations that used different amino lipids have shown to knockdown effectively genes of interest, but also triggered immune, liver and kidneys toxicities and thus could not be used for human testing.

**Figure 2:**
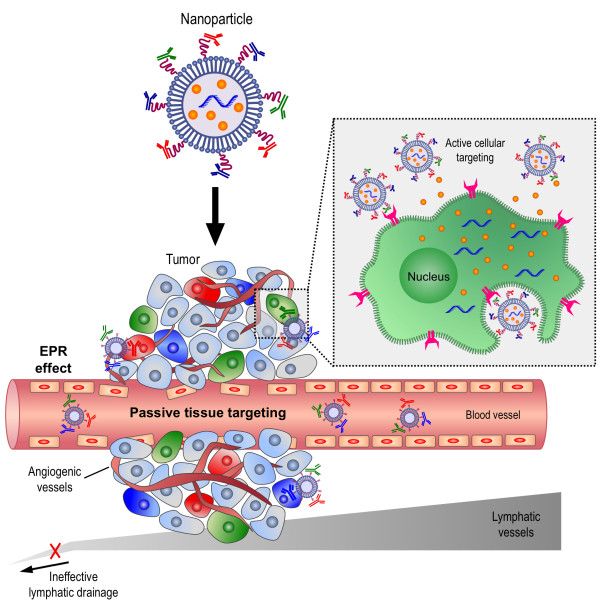
**A graphic representation of the potential mechanisms, by which nanoparticles can deliver RNAi to tumors.** Multi-functional lipid-based nanoparticles (NPs) co-encapsulated with chemotherapeutic drug (orange dots) and RNAi payloads are shown as illustrative nanoparticles. Passive tissue targeting is achieved by extravasation of nanoparticles through increased permeability of the tumor vasculature and ineffective lymphatic drainage (EPR effect). Active cellular targeting (inset) can be achieved by decorating the surface of nanoparticles with multiple targeting moieties that promote cell-specific recognition and binding. The nanoparticles can reach different tumor subpopulation concomitantly to ensure maximal therapeutic effect and release their contents in close proximity to the target cells, attach to the membrane of the cell and act as an extracellular sustained-release drug depot or internalize into the cell introducing their payload to cell cytoplasm. Red labeled cells – infiltrating macrophages that are acting as ‘nurse-like’ cells aiding the tumor by supplying survival cues to the tumor cells and nutrients for tumor growth.

Different types of adverse effects have been documented with the use of different amino lipid-based nanoparticles [34, 35, 52, 53] thus, there are some activities to form lipid-based nanoparticles with neutral charge for systemic delivery of RNAi payloads. One example is the use of 1,2-dioleoyl-sn-glycero-3-phosphatidylcholine (DOPC) which do not have PEG on their surface and entrap RNAi payloads targeted against various mRNA in ovarian cancers and melanoma [54, 55]. These lipid-based particles were shown to accumulate in cancerous tissues possibly due to the Enhanced Permeability and Retention (EPR) effect. This phenomena is based on increased fenestrations of the blood vessels in tumors caused by rapid and defective angiogenesis, and dysfunctional lymphatic drainage that retains the accumulated particles [56], see Figure 2[3].

Lipidoid (synthetic lipid-like molecules)-containing liposomes are additional siRNA delivery strategy that has been shown to induce effective genes silencing ( < 80% reduction in ApoB and Factor VII mice’s mRNAs levels) in the liver [3]. Single intravenous administration of lipidoid-containing lipid-based nanoparticles (LNPs) encapsulate ApoB-siRNA resulted in 50% decrease in the protein level 3 days and lasted up to 2 weeks after the treatment. Although no major immune toxicity was observed, increases in the levels of liver enzymes suggested a potential liver toxicity [3, 57, 58] (Figure 1).

Histidine-Lysine (HK) peptides are an additional, simple and effective RNAi delivery strategy. While lysine is important for binding the RNAi payloads, histidines stabilizes these particles and have an important role in buffering acidic endosomes, thereby enabling endosomal disruption and payload release [30]. HK peptides carrying Raf-1-siRNA or human rhomboid family-1- siRNAs induced significant silencing of target genes and suppress the progression of tumor-bearing mice in a human xenografts models [30, 59, 60].

Atelocollagen (AC) consists of a no- to minimal fraction of pepsin-digested collagen type I from calf dermis. AC is rich in positively charged amino acids such as lysine and hydroxylysine. It condenses the negatively charged RNAi payload and interacts with the plasma membrane, hence helps to incorporate the RNAi into the cells’ cytoplasm [30]. Indeed these biomaterials have not been modified to target specifically cancerous tissues, however, it have been shown that due to the EPR effect an accumulation of AC within tumor vicinity is documented in several tumor xenografts mouse models [27, 30, 61–63].

#### *Systemic active cellular delivery strategies*

siRNAs conjugated to different molecules that can aid in cellular targeting is a common strategy for active cellular delivery. One such example is the Cholesterol – siRNA conjugate [3, 30]. This delivery strategy is based on the lipoprotein to which the cholesterol-siRNAs conjugates are attached in the circulation. When the conjugates bind low density lipoprotein (LDL), the particles are mainly taken up by the liver due to the wide expression of LDL-receptors whereas when they bind to high density lipoprotein (HDL), these conjugates accumulate in the gut, the kidney, the liver and steroidogenic organs, all of which express scavenger receptor class B, type I (SR-BI) receptors, which bind HDL [3, 64]. Cholesterol-ApoB-siRNA conjugate as well as α-tocopherol [65] and lithocholic acid or lauric acid conjugated to ApoB-siRNA [66] reduced serum cholesterol and ApoB mRNA levels in the liver [3, 9].

An additional strategy in this category is the dynamic polyconjugates (DP) [3, 67]. This strategy includes membrane-active polymers that when reaching the acidic environment of the endosomes release the RNAi payloads. Using N-acetylgalactosamine, which binds to the asialoglycoprotein receptor, the DP targets preferentially hepatocytes. Like the other strategies that target the liver (e.g. SNALP), these particles have shown to effectively decreased ApoB mRNA levels in the liver when ApoB-siRNAs were used as the RNAi payloads [3].

Polyethyleneimine (PEI) complexes carrying RNAi payloads have also shown to trigger in vivo gene silencing when administrated subcutaneously into human tumors implented in nude mice. These PEI complexes composed of RGD (Arg-Gly-Asp) tripeptide that endow them specificty to integrins (adhesion receptors highly expressed on tumor cells and extracellular materix around the tumor) and coupled via PEG (aimed at longer circulation half-life, and reduced immunogenicity) to PEI (a cationic polymer that in addition to its ability to condense nucleic acids, its pH-buffering property disrupts endosomes, thus enabling to reach the cytoplasm) [3]. When RGD-PEG-PEI are complexed with RNAi payloads, they form a polyplex that target tumor vasculature and some cancerous tissues expressing integrins (α_v_β_3_ and α_v_β_5_) [68].

cyclodextrin-containing polycation (CDP) particles have been successfully used for siRNAs delivery into mice’s subcutaneous tumors [69] and have shown promise in clinical trials [70]. CDP is a polymer with a cyclic glucose backbone that when complexed with nucliec acids assembles into a colloidal 50–70 nm in diameter particle. To attain targeting capabilities, transferin (Tf)-coupled PEG is attached to the surface of the particles exploiting the upregulation of Tf receptors in many types of tumors. CDP is considered non-toxic compared with conventional cationic polymers. Safety experiments performed with non-human primates revealed that in the highest concentration tested, administration of CDP-based particles induced increase in blood urea (could be a sign of kidney toxicity), slight elevation of liver enzyme levels in the serum and a mild increase in IL-6 levels [3, 71, 72]. In addition, repeating intravenous administration of the CDP-based particles provoked the formation of antibodies to human-Tf. Despite those disadvantages, the efficacy of Tf-coupled CDP containing siRNAs for ovarian cancer treatment is evaluated nowadays in a clinical trial [3, 70, 71].

An innovative platform strategy based on a fusion protein carrier having a specific ligand (mAb fragment) fused to human trucated protamine (RNA condenser) was developed to deliver RNAi payloads in a receptor-specific manner [73] (Figure 1). Protamines are 5–8 kDa proteins and highly basic composed of 55-79% arginine residues [74]. Positively charged, protamine interacts with the negatively charged RNAi payload, hence stabilizes, neutralizes and condenses the RNAi payloads. As a proof-of-concept, anti-Her 2 (ErbB2)-human truncated protamine fusion protein in complex with siRNA significantly suppresses the growth of breast cancer cells [3, 73].

Aptamer-siRNA chimeras (Figure 1) are RNA-based nanostructures for specific delivery of siRNAs. This strategy is based on the ability of organized RNAs to bind proteins with high specificity and affinity. The chimera comprise of a targeting entity, the aptamer, and an siRNA component. These chimeras have shown to bind and deliver specifically siRNAs into human xenograft prostate cancer model. The aptamer itself bind to PSMA, a cell-surface receptor overexpressed in many types of prostate cancer cells and tumor vascular endothelium, whereas the siRNAs decrease the expression of survival genes [3, 75]. This strategy reduced adverse effects therefore these chimeras have low immunogenicity. In addition, these chimeras can be easily scaled up at a relatively low cost and posses smaller dimensions compared with that of monoclonal antibodies (<15 kDa versus 150 kDa), which should endorses improve tissue penetration [3, 30].

Various approaches of targeted amino-based LNPs serve for targeting of stellate cells in the liver. These cells are involved in the formation of scar tissue in response to liver damage such as fibrosis or as nurse-like cells in the case of a liver cancer (hepatoma) [30]. These specilized macrophages express unique receptors for retinol binding protein which uptake vitamin A. Using this approach, intravenous administration of amino-LNPs coupled to vitamin A and complexed with siRNA to a murine key fibrogenesis factor (gp46) into cirrhotic mice, knockdown specific gene in mice’ liver and resolved fibrosis [30, 76]. 1,2-dioleoyl-3-trimethylammonium-propane (DOTAP) bearing LNPs encapsulating HER2-siRNAs and containing HK peptides (to enhance the escape from the endosomes) coupled to a single-chain antibody fragment (scFv) that binds to transferrin receptors (elevated on the surface of many cancerous cells), have been targeted to tumor xenograft and inhibited tumor progression [30, 77]. Anisamid-PEG-Liposomes-polycation-DNA (anisamid-PEG-LPDs) are unilamellar amino-LNPs coated with PEG-linked anisamide (a small-molecule compound that binds sigma receptors) on their surface, and protamine-condensed mixture of siRNAs and a carrier calf thymus DNA in their core. Encapsulating EGFR-siRNA, anismaide-PEG-LPDs administrated systemically into mice bearing tumors, have been shown to increase mice’s sensitivity to chemotherapy [30, 78]. These interesting LNPs triggered the realase of cytokines in robust manner which could hamper their immediate translation into clinical practice. Yet, induction of cytokine response is not always poisonous and there are substential cases when triggering an immune response (mostly pro-inflammatory response) could augment the therapeutic response.

#### *Manipulating leukocytes with RNAi via targeted strategies*

Manipulating gene profiling in leukocytes holds great promise for drug discovery and for enhancing the development of new therapeutic modalities for leukocytes implicated diseases such as inflammation, hematological malignancies, and leukocytes-tropic viral infections. Yet, systemic delivery of RNAi to leukocytes has proven to be extremely challenging since leukocytes are intrinsically resistance to conventional transfection mechanisms and since they are dispersed in the body and thus need specific cell surface markers to be used as targeting moieties [30, 47].

siRNAs that are linked (by chemical synthesis) to a CpG oligonucleotide agonist of Toll Like Receptor (TLR)9 for targeting myeloid cells and B cells (both are key components of tumor microenvironment) that express this receptor were reported by Kortylewski et al. [79]. These conjugates knockdown the expression of the gene *stat3* by using the RNAi payload and simultaneously activated TLRs responses using its agonist. Consequently, the tumor microenvironment was changed from pro-oncogenic environment to an anti-oncogenic (by causing activation of tumor-associated immune cells and potent antitumor immune responses) [30].

An interesting strategy simillar to the protamine fusion protein startegy was developed to treat viral-tropic infections. scFv against CD7, a surface antigen present on the majority of human T cells was modified to include a Cys residue for conjugation to a 9 arginine (R) residue. The concept was that the scFv will target a specific cell surface receptor while the 9R will aid in complexing the RNAi payload. This elegant strategy was utilized for specific delivery of CCR5 (a chemokine receptor that function as co-receptor for HIV) and Vif/Tat (HIV replication proteins) -siRNAs payloads into T cells, and had shown to suppress HIV infection in humanized mice without inducing toxicity in target cells [3, 80, 81]. The same strategy was used for trying to treat dengue virus infected cells. This study utilized DC3, a 12-mer peptide that targets dendritic cells coupled to 9R. TNF-α siRNAs was used to decrease the mRNA levels. In dengue pathogenesis TNF-α plays a pivotal role and is highly conserved sequence in the viral envelope. These complexes significantly reduced virus-induced production of TNF-α and succeeded to suppress the viral replication in monocytes derived dendritic cells and macrophages in vitro. In vivo, treatment of mice with intravenous injection of DC3-9dR-complexes carried TNF-α–siRNAs, effectively suppressed this cytokine production by dendritic cells [3, 82].

The strategy we developed for targeting subsets of leukocytes is based on leukocytes’ integrins, which are the most characterized cell adhesion molecules that mediate cell-cell and cell-matrix interactions [3, 83]. Utilizing the lymphocyte function associated antigen-1 (LFA-1) integrin, which is expressed in all leukocytes’ subtypes, we developed scFv-protamine fusion proteins for selective targeting. The use of LFA-1 for targeting leukocytes is supported by its exclusive expression on leukocytes, its constitutive internalization and recycling activity and its ability to undergo activation-dependent conformational changes. Using those antibody-protamine fusion proteins we have shown specific delivery of RNAi payloads into leukocytes in vitro and in vivo. Prominently, lymphocytes activation or interferon response was not observed making this strategy a safe option for therapeutic intervention. In addition, a high-affinity conformation reagent that target LFA-1 only in activated lymphocytes was prepared and characterized [84, 85]. We showed a superior selectivity towards activated lymphocytes, which could provide a way to overcome the undesirable immune activation on bystander immune cells. Additionally, due to the prevalent of aberrant affinity modulation of integrins in a variety of leukocyte-implication diseases [86, 87], targeting the high-affinity conformation of LFA-1 is expected to be very effective therapeutic strategy [3, 47, 85].

To substantially enlarge the amount of the RNAi payload and attain a superior and more selective gene silencing, we have formed LNPs decorated with anti-integrin mAb termed ‘Integrin-targeted stabilized nanoparticles (I-tsNP)’ that successfully delivered RNAi payloads into subsets of leukocytes involved in gut inflammation [88]. These particles are made from neutral liposomes to form multilamellar vesicles (MLV) are extruded to ~80 nm in diameter unilamellar vesicles (ULV) are covalently coated with hyaluronan (HA), a naturally-occuring glycosaminoglycan, for stabilization of the particles during RNAi encapsulation. HA also prolong the in vivo half-life in the circulation. Targeting capabilities is formed by coupling a mAb against the β_7_ integrin (which is highly expressed in gut leukocytes) to the HA [89]. Utilizing this strategy, we found a new, unknown role for cyclin D1 as a potential anti-inflammatory therapeutic target controlling some of the pro-inflammatory cytokines. These leukocytes-targeted- LNPs offer one of the safest platforms for nucleic acids delivery, preventing cytokine induction, interferon response and complement activation as well as liver and kidneys toxicities. This strategy was also utilized for delivery of CCR5 -siRNAs to human lymphocytes and monocytes with an anti-LFA-1 mAb. LFA-1 I-tsNPs entrapping CCR5-siRNAs protected mice from HIV challenge [90] and did not induce secretion of TNF-α (one of the major players in inflammation) nor did it trigger interferon response, hence reinforces the vast potential for clinical translation. Since this strategy is a platform, it is very likely that by exchanging the mAb on the surface, for example, against B cell malignancies (anti-CD19 or CD20) it will be possible to target many types of B-cells malignancies such as mantle cell lymphoma (MCL), and chronic lymphocytic leukemia (CLL) in a specific and efficient manner. In addition, by using activated –dependent conformational reagent such as AL-57 [85] – it will be possible to target activated cells such as multiple myeloma cells (MM) while leaving naïve B cells untouched.

### Clinical translation of siRNA nanomedicines

Several RNAi-based clinical trials utilize naked RNAi molecules with local delivery (Table 1), yet, a great promise for the entire RNAi field is based on systemic administration and vast progress has been made using non-human primate models [49, 91–95].

**Table 1:** **Novel siRNAs –based drugs in clinical trials (adapted and revised with permission from** [9, 96]**)**

Candidate name	Disease	Target	Delivery system	Phase	Year
Bevasiranib	AMD	VEGF	Local - intravitreal needle injection.	lll - was interupted in 2009	2004
Cand5	AMD, DME	VEGF	Local - intravitreal needle injection.	ll	2004
ALN-RSV01	RSV infection	Virial RNA	Local - inhalation of unformulated siRNAs.	ll	2005
DGFi	Acute kidney injury, delayed graft function	p53	Systematic - naked siRNA	ll	2007
TD101	Pachyonychia congenita	Mutant keratin (K6a)	Local - intradermal needle injection	l	2008
QPI-2007	Chronic nerve atrophy, nonarteritic ischemic optic neurophaty	Caspace 2	Local - intravitreal needle injection	l	2009
siG12D LODER	Operable pancreatic ductal adenocarcinoma	Mutated KRAS	Local - drug elution	l	2010
CALAA-01	Metastatic solid tumors	RRM2	Systematic - CDP NPs	l	2008
ALN-VSP02	Liver cancer, cancer with liver involvement	VEGF, KSP	Systematic - SNALP liposomes (hypatocytes)	l	2008
Atu027	Advanced solid tumors	PKN3	Systematic - AtuPLEX lipoplex (vascular endothelial cells)	l	2009
TKM-ApoB	Hypercolesterolemia	ApoB	Systematic - SNALP liposomes (hypatocytes)	l	2009
ALN-TTR01	ATTR	TTR	Systematic - SNALP liposomes (hypatocytes)	l	2009
TKM-PLK1	Solids cancers and lymphoma	PLK1	Systematic - SNALP liposomes (solid tumors)	l	2010
ALN-PCS02	Hypercolesterolemia	PCSK9	Systematic - SNALP liposomes (solid tumors)	l	2011
TKM-EBOLA	Ebola infection (biodefence)	Viral RNA polymerase	Systemic - SNALP liposomes (hypotocytes and phagocytes)	l	2011

*Abbreviations*: *ApoB* Apolipoprotein B, *AMD* age-related macular edema, *ATTR* transthyretin (TTR)-mediated amyloidosis, *CDP* Cyclodextrin polycation, *DME* diabetic macular edema, *KSP* kinesin spindle protein, *NP* nanoparticle, *PCSK9* proprotein convertase subtilisin/kexin type 9, *PKN3* protein kinase N3, *PLK1* polo-like kinase 1, *RRM2* ribonucleotide reductase subunit 2, *RSV* respiratory syncytial virus, *siRNA* small interfering RNA, *SNALP* stable nucleic acid lipid particles, *VEGF* vascular endothelial growth factor.

The first sets of clinical evaluations introduced naked siRNAs to patients suffering from age-related macular degeneration (AMD) and diabetic macular edema (DME). The administration of these siRNAs was done intraviteral and the siRNAs were against vascular endothelial growth factor (VEGF). Other local strategies to deliver RNAi payloads were conducted, but many of these strategies were ended because of safety issues [96, 97]. Since then, tremendous efforts were done to refine and advance these local strategies and adjust them for systemic administration (Table 1) [9].

The first-in-human systemic administration trial engaging the use of targeted NPs for RNAi payloads to solid tumors – named CALAA-01, was conducted in 2008 [70, 71]. CALAA-01 product is based on the Tf-CDP NPs previously described in mice and non-human primates [69, 94], which form a 70 nm in diameter particle, formulating the an anti- ribonucleotide reductase subunit 2 (RRM2) siRNAs [71]. Biopsies taken from melanoma patients that were given intravenous injections of Tf-CDP-NPs showed intracellular accumulation of the Tf-CDP in the cancerous tissues and not in neighboring tissues in a dose-dependent manner. Moreover, reduction in both *RRM2* mRNA and RRM2 protein levels after several cycles of dosing was demonstrated providing promise and hope to the field of systemic RNAi delivery strategies [70].

Meanwhile several other systemic delivery strategies entered the clinical trials, six are based on SNALP strategy [9, 48]. A remarkable achievement was done in 2008, when SNALP-based systems formulated two different RNAi payloads against VEGF and kinesin spindle protein (KSP) in a single vehicle and systemically given to patients with liver cancer and transthyretin (TTR)-mediated amyloidosis (ATTR) [48]. This therapy was well tolerated and induced anti-tumor effects in most patients. In 2009, three novel drugs entered clinical evaluation, two of which, ALN-TTR01 and TKM-ApoB were SNALP-based delivery systems targeting hepatocytes and formulated with anti- Transthyretin (TTR) and anti-ApoB siRNAs in ATTR and hypercholesterolemia patients, respectively [9]. A single administration of ALN-TTR01 resulted in a significant reduction in amyloid deposits in the desired tissues as well as in the serum level of the TTR protein in a dose-dependent manner [9]. TKM-ApoB trial was terminated possibly since only some patients responded. In addition, several patients experienced flu-like syndrome usually known as a cytokine storm at the highest dose administrated. A year later, a novel formulation based on the SNALP system and targeting polo-like kinase 1 (PLK1) was evaluated in patients with advanced solid tumors and lymphoma that were unaffected by other conservative treatments. This therapeutic strategy is aimed to suppress cell-cycle progression and induce apoptosis in the cancerous tissue.

Another intriguing therapeutic strategy, which was clinically tested in patients with advanced colon cancer, targets Protein Kinase N3 (PKN3), an important mediator of the PI3K pathway. To deliver PKM3-siRNAs, Atu027 lipoplexes were used. Atu027 was reported to activate the complement system in clinical trials, and thus needs to be further optimized for future clinical testing. A novel strategy targeted against Ebola virus (TKM-EBOLA) and a strategy to reduce hypercholestrolemia (ALN-PCS02), both SNALP-based formulations entered clinical evaluation in 2011 (Table 1) [9, 97].

## Conclusion

The promise of RNAi to manipulate the function of virtually any gene in the human genome open new avenues to personalized the treatment of many types of diseases. This potential coupled with the development of selective and safe nano-scale delivery strategies generated the basis for targeted therapy in many types of illnesses such as cancer (solid and blood), inflammation, and viral infections. Yet, the lack of in deep understanding of molecular pathways in health and diseases together with general toxicities related to engineered nanomedicines can hamper the full potential for precision RNAi-based medicine and restrain the development of clinically approved novel RNAi modalities.

Although the great progress achieved towards personalizing disease care, the current personalized therapies targets a single specific molecular marker on tumor cells and thus neglecting the vast heterogeneity presents within the primary tumor, between different metastases, within each metastasis and between tumors of different patients. Consequently, current treatment usually results in apparent partial or complete responses, which in most cases follow by a resistant tumor relapse and mortality of the patients. Therefore, personalized treatment requires multi-layer characterization of the molecular signatures of tumor cell subpopulations together with individualized pharmacotyping (identify specific sequence alternations in potential therapeutic targets, enzymes that metabolized drugs as well as drug transporters that may alter the efficacy, specificity and sides effects) to achieve maximal efficacy.

We envision that the growing efforts for fine-tuning NPs design, optimizing intracellular payload release, successful approaches in minimizing adverse effects, and functionalizing the NPs with selective targeting moieties, along with the comprehensive understanding of our genome and transcriptome, would collectively tile the way for specific, safe and targeted NPs that carry RNAi payloads for precision medicine.
